# Theoretical study on the hydrogen distribution and diffusion at the PuO_2_/α-Pu_2_O_3_ interface

**DOI:** 10.1039/d4ra02257b

**Published:** 2024-05-22

**Authors:** Huang Huang, Min Zhu, Ming Guo, Longxian Li, Yan Li

**Affiliations:** a Naval University of Engineering Wuhan 430033 China

## Abstract

The interface is a region in the crystal that significantly changes various characteristics. There must be an interface between oxides of different valence states in the surface oxide layer of plutonium. In this work, a first principles approach based on DFT was used to study the hydrogen distribution and diffusion at the PuO_2_/α-Pu_2_O_3_ interface systematically. Our research reveals that at the interface, hydrogen can be captured by the O atoms of PuO_2_ and by the oxygen vacancies (OVs) of α-Pu_2_O_3_, and the capture of OVs is more energetically advantageous. On the PuO_2_ side, the cost of H atom diffusion towards the interface gradually increases. On the α-Pu_2_O_3_ side, the cost of H atoms diffusing inward from the interface gradually increases. OVs that already contain H atoms are more conducive to capturing H atoms. The formation of the interface has little effect on the hydrogen capture ability of O in PuO_2_, but it will reduce the capture ability of OVs in α-Pu_2_O_3_. Overall, the formation of interfaces has no disruptive impact on the behavior of hydrogen in the two plutonium oxides. This is closely related to the fact that α-Pu_2_O_3_ originates from PuO_2_ under anaerobic conditions. The difference in hydrogen behavior comes from the changes in the atomic environment and ion valence state caused by the OVs. This work supports further understanding of the behavior of hydrogen in plutonium oxides and provides a reference for further research on plutonium corrosion prevention.

## Abstract 2

The interface will not significantly change the doping characteristics of hydrogen in PuO_2_ and α-Pu_2_O_3_, acting as a buffer layer.

## Graphical abstract

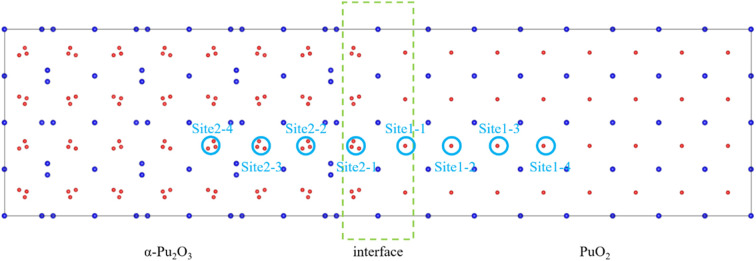

## 1 Introduction

Plutonium is an indispensable material in the nuclear industry. In actinide elements, plutonium's complex 5f electronic state brings it active physical and chemical properties.^1,2^ Naturally, in the air, the surface of plutonium will be rapidly oxidized, forming a complex oxide layer.^3–5^ It is generally believed that the outermost layer of the oxide layer is PuO_2_, and the inner layer is Pu_2_O_3_, forming a sandwich structure of PuO_2_/Pu_2_O_3_/Pu.^6–8^ Compared to oxidation corrosion, hydrogenation corrosion poses a serious threat to plutonium's safe handling and storage. The presence of oxide film on the surface of plutonium leads to four stages of hydrogen corrosion of plutonium: induction, nucleation or acceleration, bulk hydriding, and termination.^9–11^ The induction period mainly involves the interaction between the hydrogen and oxide layer, which is time-consuming and controllable.^12^ The induction period is key to the entire hydrogenation corrosion process.

Experimental studies have shown that PuO_2_ can effectively prevent the entry of hydrogen. The complete PuO_2_ layer is the first barrier for plutonium hydrogenation corrosion. However, the Pu_2_O_3_ layer cannot effectively prevent hydrogen erosion and provides the main hydrogen nucleation sites.^13,14^ Based on the basic understanding of experiments, people have conducted mechanism research on related issues from a microscopic perspective. Sun^15^ first analyzed the collision-induced dissociation of hydrogen molecules on intact and defective PuO_2_ layers, finding that hydrogen is more prone to dissociation in defective systems. Only dissociated hydrogen can penetrate the PuO_2_. Yu's^16,17^ study about the adsorption mechanism of H_2_ and H atoms on the surface of PuO_2_ (110) shows that the dissociation barrier of H_2_ is 0.48 eV. H atoms tend to exist on the outer surface rather than migrating to the subsurface.

In addition to the surface, the characteristics of hydrogen in the plutonium oxide phase have also received some attention. In Ao's study about the existence state of hydrogen in the PuO_2_ bulk phase, it was found that hydrogen is either insoluble or just at the edge of dissolution.^18–20^ This low solubility leads to rapid accumulation of hydrogen in the defect area and rapid diffusion in the PuO_2_ layer. Zhang *et al.*^21,22^ conducted a comparative study on the states of hydrogen in PuO_2_ and Pu_2_O_3_, concluding that it is very difficult for H to dissolve in intact PuO_2_. In addition, they also found that H is the preferred state of existence in PuO_2_, but H atoms spontaneously recombine in Pu_2_O_3_. It is proposed that the high endothermic adsorption and dissolution properties of hydrogen in PuO_2_ are the primary mechanism for hydrogen inhibition rather than hindering the diffusion kinetics of H, which also confirms the relevant conclusions of Ao. Using molecular dynamics methods, Tang^23^ analyzed the diffusion behavior of hydrogen in oxygen-saturated (OS) and oxygen-unsaturated (OU) plutonium oxides. The results showed that due to the diffusion trap effect of OVs, the diffusion coefficient of OU PuO_2_ was lower than that of OS PuO_2_. Using a similar method, Tang^24^ also analyzed the different roles played by PuO_2_ and Pu_2_O_3_ in hydrogen inhibition. The hydrogen inhibition effect of PuO_2_ is mainly due to the capture of H atoms by lattice oxygen to form hydroxyl groups. For Pu_2_O_3_, when the hydrogen concentration is low, hydrogen erosion can be prevented because OVs act as traps for hydrogen migration. However, when the hydrogen concentration is high enough, it cannot effectively resist hydrogenation corrosion. The above studies indicate that in the oxide layer formed on the surface of plutonium exposed to air, there are significant differences in the hydrogen-blocking mechanisms between the two typical oxides, PuO_2_ and Pu_2_O_3_. In the sandwich structure of PuO_2_/Pu_2_O_3_/Pu, an interface exists between PuO_2_/Pu_2_O_3_, and the behavior of hydrogen at this interface is worth paying attention to.

This work systematically investigated the hydrogen distribution and diffusion behaviors in PuO_2_, α-Pu_2_O_3,_ and their interface with the first-principles calculations. Our objective is to elucidate the role of the interface in the interaction process between hydrogen plutonium oxide. The rest of this paper is organized as follows. Our methodological approach and modeling are presented in Section 2, and our results are discussed in Section 3. Section 4 contains our main conclusions.

## 2 Methodology and modeling

### 2.1 Methodology

First-principles calculations are conducted using the VASP (Vienna *Ab initio* Simulation Package) software package.^25^ The correlation properties of electronic exchange are described by the GGA-PBE (Perdew–Burke–Ernzerhof of generalized gradient approximation) functional.^26,27^ A plane-wave kinetic energy cutoff of 520 eV is shown to give an accurate convergence of total energies. The 6s^2^7s^2^6p^6^6d^2^5f^4^ electrons of Pu and the 2s^2^2p^4^ electrons of O participate in the calculation as valence electrons.

The Hubbard model is used within the DFT + *U* method in the Dudarev formalism to treat strong on-site Coulomb interaction.^28^ An effective *U* (*U*_eff_ = *U* − *J*; *i.e.*, the difference between the Coulomb *U* and exchange *J* parameters, hereafter referred to as *U*) value of 4 eV is selected for the 5f electrons of Pu and U, according to our previous calculations^29,30^ and other computational experience.^21,22,31–36^ According to experimental and theoretical calculations (DFT + *U*), the ground states of Pu_2_O_3_ and PuO_2_ are set to antiferromagnetic states (AFM).^28,37^ Correction of van der Waals forces between H, H_2_, and plutonium oxide matrix using DFT-D3 method.^38,39^ The Brillouin zone selects 6 × 6 × 6 and 6 × 6 × 2 Monkhorst–Pack^40^ lattice points for single type oxide model and interface model respectively, with an energy convergence standard of 0.01 eV Å^−1^.

The calculation of transition states adopts the CINEB^41,42^ method (clipping image nudge elastic band method). The defect formation energy (*E*_a_) of the particle in the plutonium oxide is expressed as*E*_a_ = *E*_basement+P_ − *E*_basement_ − *E*_P_where *E*_basement+P_ shows the total energy of the adsorption system, *E*_basement_ means the total energy of the basement. *E*_P_ means the total energy of the free particle. For the simultaneous adsorption of multiple particles of the same type, the average energy (*E*_a-ave_) must be considered. The average energy is expressed as*E*_a-ave_ = (*E*_basement+P_ − *E*_basement_ − *nE*_P_)/*n*

A negative of *E*_a_ means heat release and spontaneous, and *vice versa*. *n* means the number of particles.

### 2.2 Modeling

We first optimize the structure of PuO_2_ single crystal cells, our calculation result for *a*_0_ is 5.432 Å, which differs from the experimental value (5.396 Å) by 0.66%.

Under hypoxic conditions, PuO_2_ can be reduced to α-Pu_2_O_3_, which has a similar cubic structure. In the structure of α-Pu_2_O_3_, O atoms occupy the 48e sites, while Pu atoms occupy the 24d and 8a sites. Based on the 2 × 2 × 2 supercell of PuO_2_, 16 O atoms are removed from the 16c (0.25, 0.25, 0.25) sites, and structural relaxation is performed to obtain the α-Pu_2_O_3_ single crystal cell. Our calculation result for *a*_0_ is 11.20 Å, which differs from the experimental value (10.98 Å) by 2.2%.

**Fig. 1:**
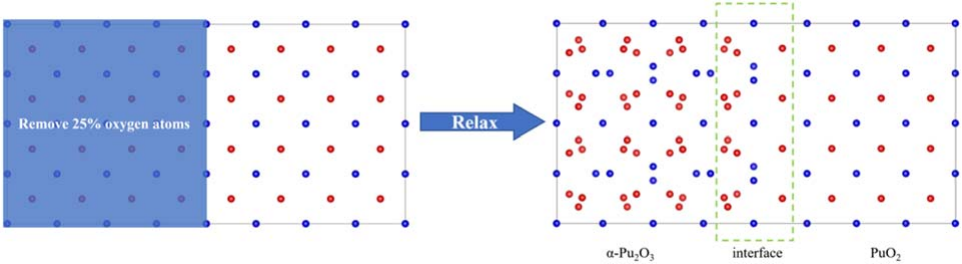
Approach 1 for obtaining interface models. Red spheres are O atoms, and blue spheres are plutonium atoms.

**Fig. 2:**
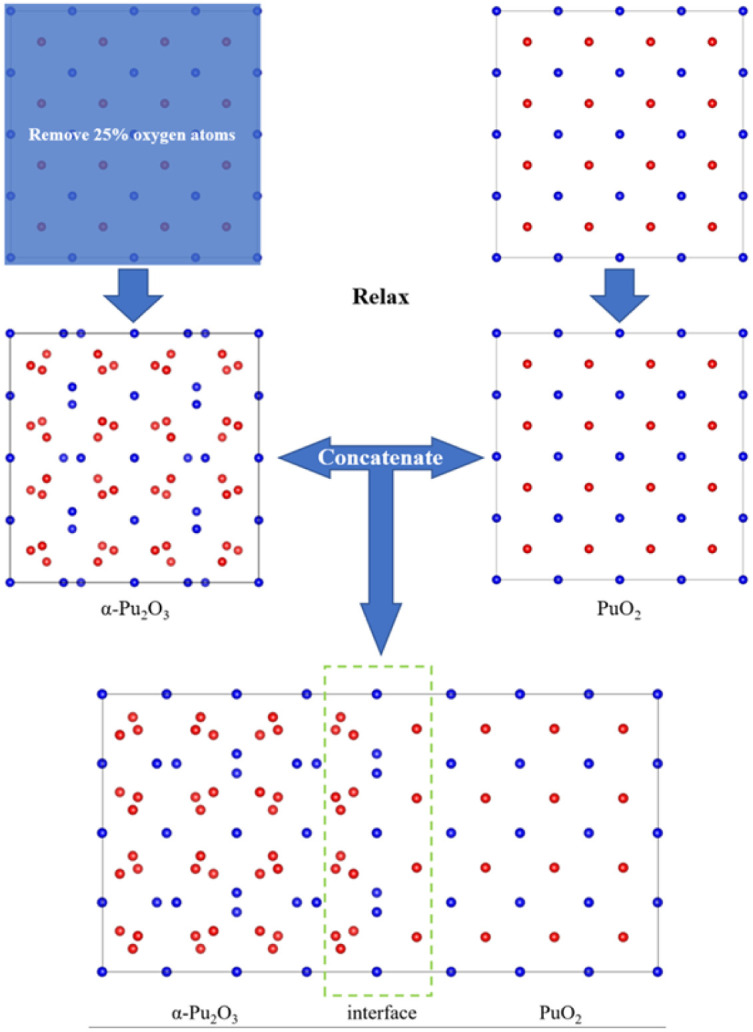
Approach 2 for obtaining interface models.

Based on the relationship between the 2 × 2 × 2 supercell of PuO_2_ and the single crystal cell of Pu_2_O_3_ mentioned above, two approaches can be used to construct the PuO_2_/α-Pu_2_O_3_ interface model:

(1) Construct a supercell model of PuO_2_ by removing O atoms from the corresponding sites in half of the model and constructing it as α-Pu_2_O_3_, followed by relaxation (Fig. 1).

(2) Firstly, a supercell model of PuO_2_ is constructed for relaxation, followed by removing O atoms at 16 corresponding sites and relaxation to obtain an α-Pu_2_O_3_ model. The relaxed PuO_2_ and α-Pu_2_O_3_ cell models are concatenated, and then the concatenated model is relaxed (Fig. 2).

After calculation, we found that the models obtained by the two approaches have slight differences in size and atomic configuration. In contrast, the first approach is more in line with PuO_2_ being reduced to α-Pu_2_O_3_ due to hypoxia.

**Fig. 3:**
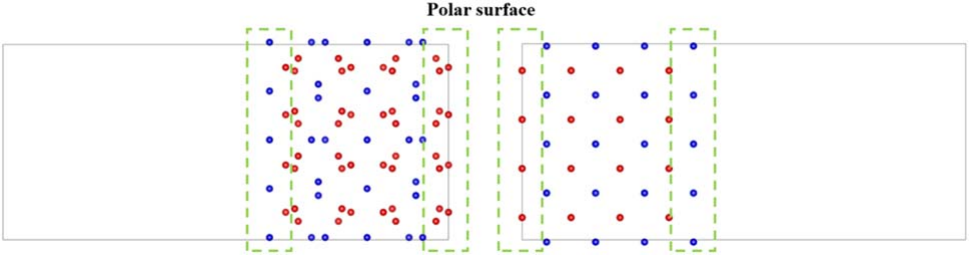
Polar surface caused by vacuum layer.

There is still a question about building a vacuum layer during model construction. If a vacuum layer is constructed, it is necessary to analyze the exposed surface. The surface exposed by the model obtained through the above two approaches is (100) surface (as shown in Fig. 3). The PuO_2_ (100) surface is polar and needs to be treated to improve stability.^43^ If a vacuum layer is not constructed, mutual contamination exists between interfaces in periodic structures. Tang^44^ chose to avoid exposing polar surfaces in his research on oxygen atom diffusion. Based on this approach, this work considers increasing the model's thickness to avoid exposing polar surfaces while minimizing mutual contamination between interfaces.

**Fig. 4:**
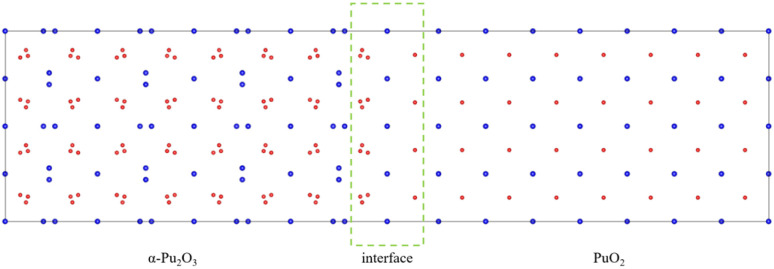
PuO_2_/α-Pu_2_O_3_ interface model.

Considering the various aspects of interface model construction mentioned above, we used approach 1 to construct an interface model with sufficient thickness without a vacuum layer, as shown in Fig. 4.

## 3 Results and discussion

### 3.1 Properties of PuO_2_/α-Pu_2_O_3_ interface

**Fig. 5:**
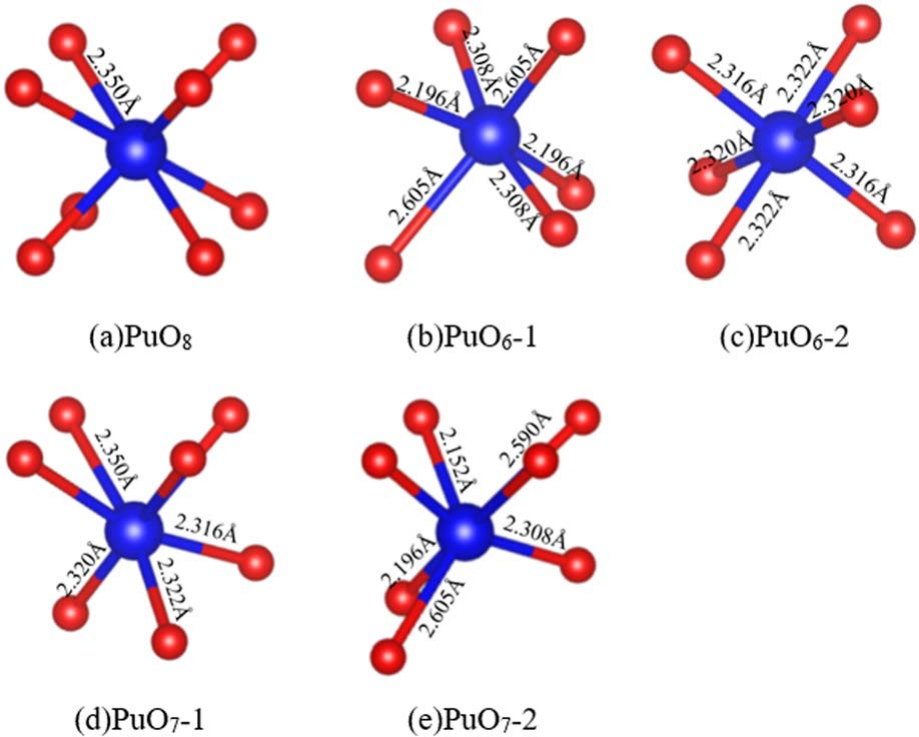
PuO_2_/α-Pu_2_O_3_ interface model. (a) Perfect PuO_8_ cube. (b) PuO_6_-1. (c) PuO_6_-2. (d) PuO_7_-1. (e) PuO_7_-2.

The environment around an atom significantly impacts its exhibited properties, especially for Pu atoms with wandering delocalized and localized 5f electrons. Analyzing the coordination of O atoms around Pu atoms is significant for understanding the overall interface characteristics. The O atom coordination number around the Pu atom in PuO_2_ is eight (4 + 4), with a distance of 2.35 Å, forming a PuO_8_ cube, as shown in Fig. 5(a). The O atom coordination number around the Pu atom in α-Pu_2_O_3_ is six (3 + 3), forming PuO_6_, and there are two states in coordination configuration, as shown in Fig. 5(b) and (c). The O atom coordination number around the Pu atom at the interface is seven (3 + 4), forming PuO_7_, and there are also two coordination configurations in different states, as shown in Fig. 5(d) and (e).

The Pu^4+^ ions in PuO_2_ are difficult to further oxidize to higher valence states. The Pu^3+^ ions in PuO_2_ are more difficult to further reduce to lower valence states.^45–47^ For PuO_2_, each OV produces the two nearest Pu^3+^ ions, similar to the microscopic description of the two electrons left behind when forming OVs in CeO_2_.^48^ At the interface, there are four OVs on the side of α-Pu_2_O_3_, which can generate eight Pu^3+^ ions, of which four belong to the inside of the interface, and four belong to the outside of the interface. The interface contains eight Pu ions, half of which are Pu^4+^ and half are Pu^3+^.

**Fig. 6:**
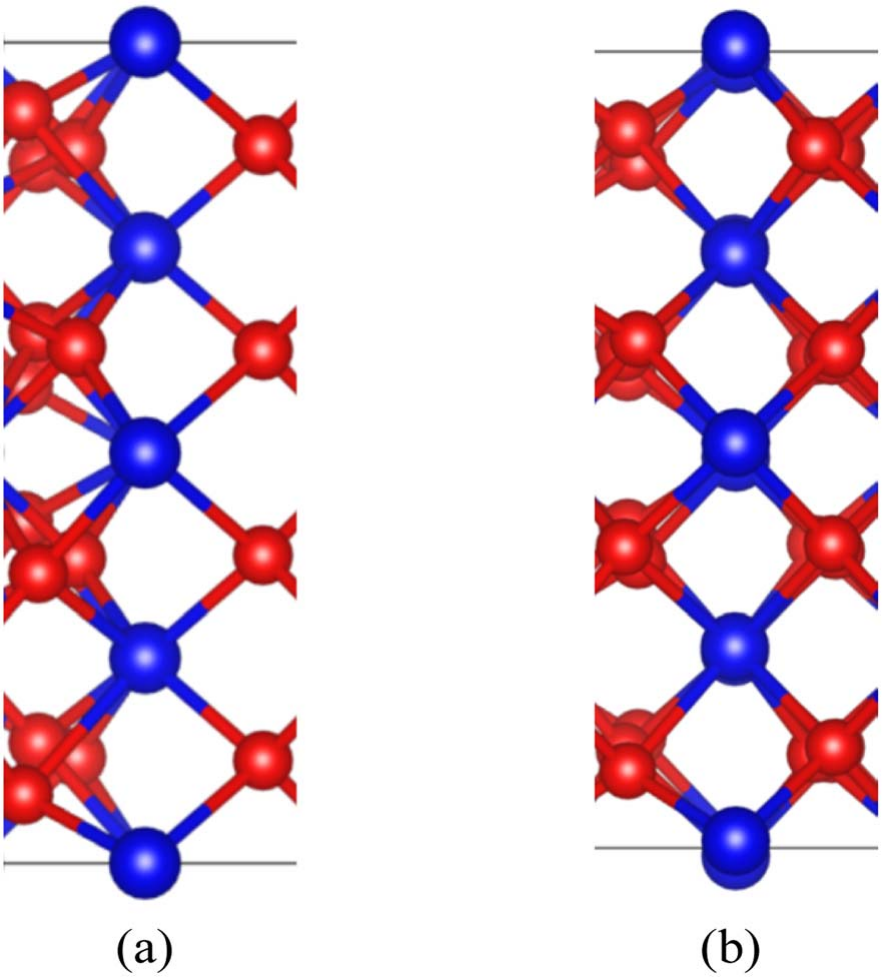
Structural distortion at the interface. (a) Configuration before structural optimization. (b) Configuration after structural optimization.

**Fig. 7:**
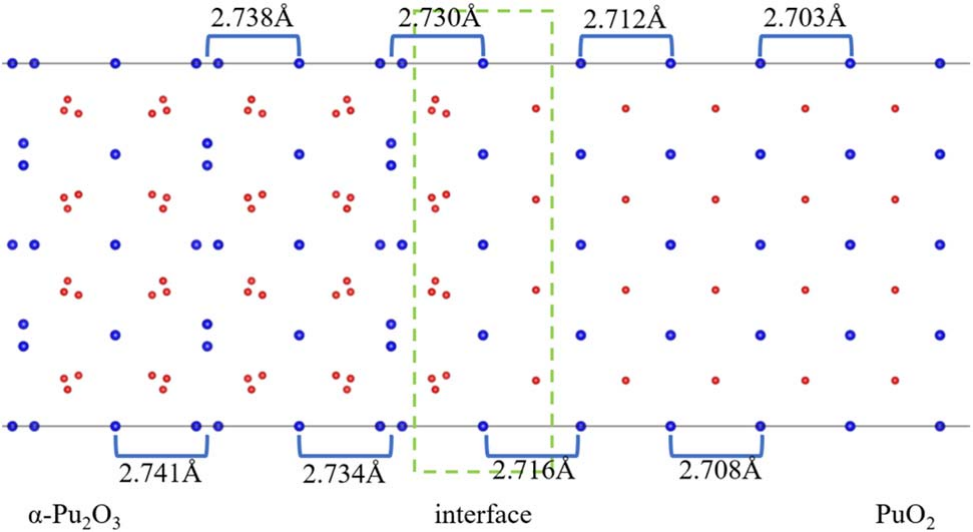
Pu atoms interlayer spacing.

After the interface's formation, relaxation will impact the atomic environment at and near the interface. Compared with before and after structural optimization, the atomic structure near the interface underwent distortion, as shown in Fig. 6. Of particular concern is the distance between each layer of Pu atoms, as shown in Fig. 7. It can be observed that the closer to the interface, the greater the interlayer spacing of Pu atoms on the side of PuO_2_, the smaller the interlayer spacing on the side of α-Pu_2_O_3_.

### 3.2 Hydrogen distribution in the PuO_2_ and α-Pu_2_O_3_

**Fig. 8:**
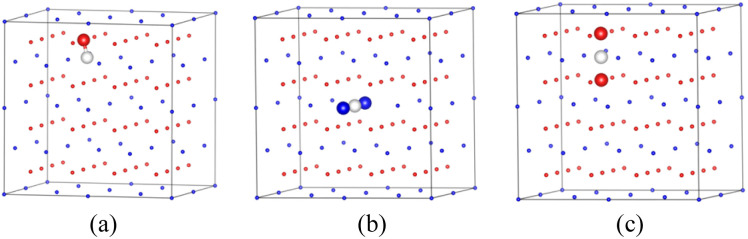
Incorporation site for H in PuO_2_. (a) First nearest-neighboring oxygen. (b) Octahedral interstitial site. (c) Two oxygen interstitial site.

Firstly, we analyze the distribution of individual hydrogen in the PuO_2_ and α-Pu_2_O_3_ before analyzing the behavior at the interface. For PuO_2_, Pu^4+^ cannot be further oxidized.^18^ After H atoms are incorporated, they can only combine with O atoms as reducing agents to form (OH)^−1^. Zhang's research also confirms that the stable existence of hydrogen in PuO_2_ is captured by O atoms in atomic form.^21^ For verification, we calculated the defect formation energy of a single H atom incorporated in the first nearest-neighboring oxygen, octahedral interstitial site, and two oxygen interstitial site, respectively (Fig. 8). The defect formation energies are 0.89 eV, 1.79 eV, and 2.11 eV, respectively, and each oxygen can capture up to four H atoms, which is consistent with the previous research.^21^

For α-Pu_2_O_3_, there seems to be some controversy over the stable state of hydrogen in it. Zhang thought that H prefers to bind to O-anion according to incorporation energies.^22^ Tang's molecular dynamics calculation results indicate that no hydroxyl was formed, but a favorable and stable capturing effect from the OV was found.^24^ To determine the state of hydrogen in α-Pu_2_O_3_ within our theoretical system, we calculated the defect formation energy of a single H atom incorporated in an OV and first nearest-neighboring oxygen. From the perspective of defect formation energy, H atoms are more likely to be captured by OVs.

We are also curious about how many H atoms each OV can accommodate. Table 1 shows the formation energy of defects where different numbers of H atoms are captured in the same OV. Combining two H atoms leads to a significant decrease in formation energy, indicating that hydrogen molecules embedded in OVs do not interact significantly with plutonium or oxygen in the surrounding environment. The lower the impact of impurities on the system, the lower the formation energy. The two hydrogen atoms doped with oxygen vacancies in molecular form have a relatively small impact on the system, resulting in a lower formation energy. When the number of H atoms reaches five, there is a sudden increase in defect formation energy. This indicates that for an OV, four H atoms are the upper limit it can accommodate.

**Table 1:** Incorporation energy for H in OV. The energy variation represents the change in the total formation energy of all hydrogen atoms caused by adding a doped hydrogen atom

Number of H	1	2	3	4	5
Formation energy (eV per H)	0.97	0.32	0.61	0.43	1.12
Energy variation (eV)	0.97	−0.33	1.19	−0.11	3.88

### 3.3 Hydrogen distribution in the PuO_2_/α-Pu_2_O_3_ interface

**Fig. 9:**
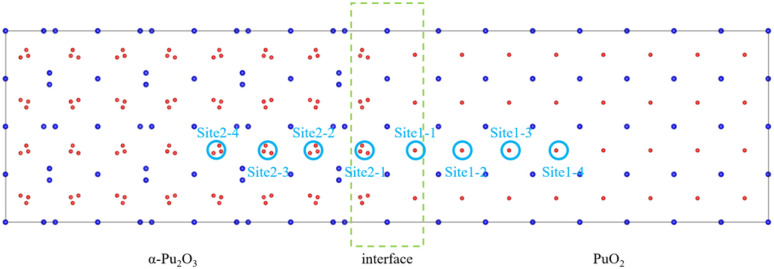
Incorporation site for H in the PuO_2_/α-Pu_2_O_3_ interface.

H atoms in PuO_2_ tend to be captured by O atoms to form hydroxyl groups, and in α-Pu_2_O_3_ tend to be trapped by OVs. Based on this, select the site near the oxygen atom on the side of PuO_2_ (hereinafter referred to as site 1–1) and the OV on the side of α-Pu_2_O_3_ (hereinafter referred to as site 2–1) as potential existence sites for H atom, as shown in Fig. 9. After sufficient relaxation, H atoms still remain near O atom or in OV, indicating that both sites are stable capture points for H atoms. The defect formation energies of H atoms at these two sites are 1.32 eV and 1.17 eV, respectively. The incorporations of H atoms at the interface are endothermic processes. H is more inclined to be absorbed by OV in α-Pu_2_O_3_.

When near the O atom on the side of PuO_2_ in the interface, the distance between H and O atoms is about 0.995 Å, and incorporating H atoms increases the system's volume by 7 Å^3^. When in the OV on the side of α-Pu_2_O_3_, incorporating H atoms decreases the system's volume by 2 Å^3^. The lattice volume is closely related to the chemical bonds in the system. Intuitively speaking, adding atoms will increase the system's volume, known as the spatial effect. The chemical bond formed between the doped atoms and the substrate will reduce the system's volume, called the bonding effect. The volume change of the system after the impurity's incorporation depends on the mutual cancellation of spatial and bonding effects. When the crystal is at site 1–1, hydrogen acts as a reducing agent, promoting the reduction of low valent Pu with a larger volume in the system. The spatial effect dominates, leading to an increase in the system's volume. When the crystal is at site 2–1, hydrogen acts as an oxidant to promote the high valence Pu with a smaller volume at the oxidation site in the system, and the bonding effect dominates, leading to a decrease in the system's volume. Using hydrogen as a probe can detect the characteristics of Pu atoms at the interface, that is, Pu atoms at the interface can be reduced and oxidized.

Through the analysis of interface characteristics above, it is found that the formation of interfaces not only affects the atomic environment at the interface itself, resulting in changes in hydrogen behavior, but also affects the atomic environment near the interface, which also causes changes in hydrogen behavior. The incorporation energies of H atoms at six sites outside the interface, located near the O atoms on the PuO_2_ side and at the OVs on the α-Pu_2_O_3_ side, were calculated to analyze the area affected by the interface. The incorporation energy of each point is shown in Table 2. On the PuO_2_ side, as the distance between the site and the interface increases, the energy required for incorporation also increases. On the side of α-Pu_2_O_3_, the energy required for incorporation decreases as the distance increases. The main reason is that the closer to the interface on the side of PuO_2_, the longer the bond length for Pu–O, and the less oxidation ability of O ions occupied by Pu, which can free up more oxidation ability to interact with adsorbed H atoms. As a comparison, on the side of α-Pu_2_O_3_, the farther the site is from the interface, the stronger the reducing ability of the surrounding Pu, which makes H atoms more easily reduced as oxidants and captured by OVs.

Incorporation energy for every site

**Table:** 

Site	Site 1–1	Site 1–2	Site 1–3	Site 1–4
Incorporation energy (eV)	1.32	1.36	1.41	1.43

**Table:** 

Site	Site 2–1	Site 2–2	Site 2–3	Site 2–4
Incorporation energy (eV)	1.17	1.12	1.06	1.03

### 3.4 Hydrogen diffusion in the PuO_2_/α-Pu_2_O_3_ interface

For the H atom, diffusing from PuO_2_ to α-Pu_2_O_3_ has seven processes. The energy barriers in each process are shown in Table 3. Overall, the diffusion energy barrier for H atoms on the PuO_2_ side is relatively lower than on the α-Pu_2_O_3_. From the changing trend, the closer the H atoms are to the interface on the side of PuO_2_, the higher the diffusion energy barrier, and the more difficult diffusion. There are two reasons for this situation: firstly, the formation of the interface leads to an increase in the distance between system atoms near the interface (compared to pure PuO_2_); secondly, the enhanced oxidation ability of O atoms leads to a tighter binding with H. On the side of α-Pu_2_O_3_, the farther the H atoms are away from the interface, the higher the diffusion energy barrier, and the more difficult it is for diffusion to occur. Two factors play a role: first, the formation of the interface leads to a decrease in the distance between atoms in the system near the interface (compared to pure α-Pu_2_O_3_); second is that the closer the Pu atom is to the interface, the smaller the radius. During the diffusion of H atoms from one OV to the following OV, they need to “squeeze” through the area formed by the Pu atoms. The smaller the Pu atom radius, the easier this process occurs.

Energy barriers for diffusion

**Table:** 

Process	Site 1–4 → site 1–3	Site 1–3 → site 1–2	Site 1–2 → site 1–1	Site 1–1 → site 2–1
Energy barrier (eV)	0.45	0.51	0.63	0.59

**Table:** 

Process	Site 2–1 → site 2–2	Site 2–2 → site 2–3	Site 2–3 → site 2–4
Energy barrier (eV)	0.64	0.66	0.69

**Fig. 10:**
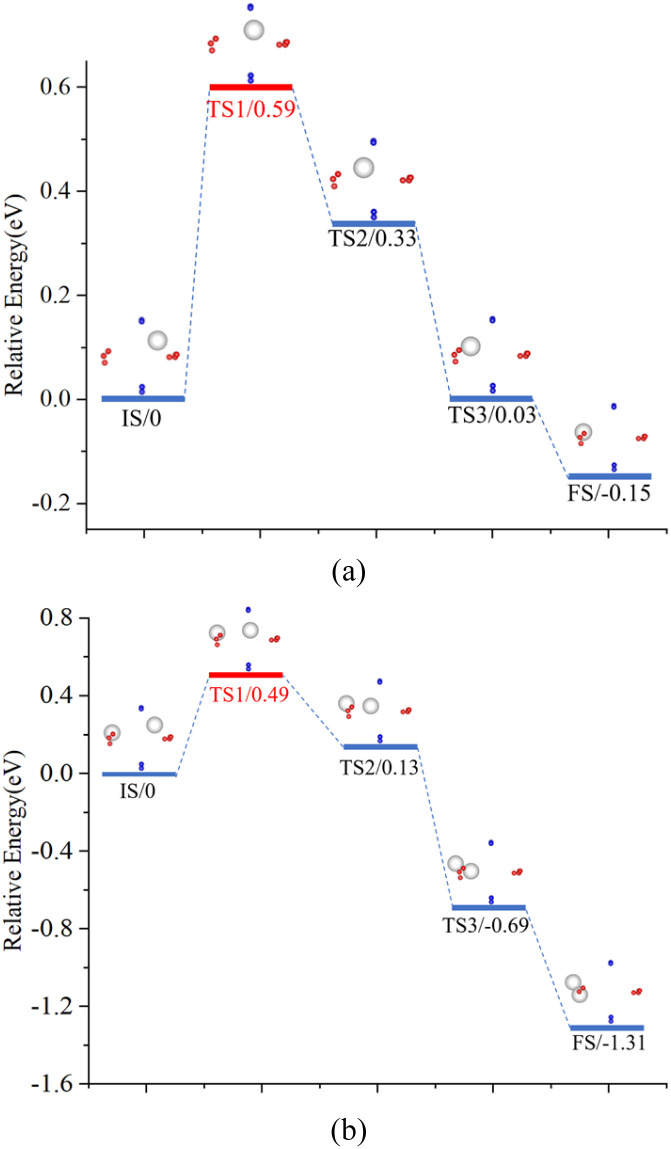
Diffusion behavior for H. The IS means initial state, the TS means transition state and the FS means final state. (a) Route 1. (b) Route 2.

A particular point requires special attention, where H atoms diffuse from Site 1–1 to site 2–1. During this process, the H atom is captured by the O atom and diffuses to be captured by OV. According to the previous analysis, hydrogen exists in an atomic state in PuO_2_ and a molecular state (or possibly in an atomic state) in α-Pu_2_O_3_. Multiple H atoms may be diffusing from PuO_2_ to α-Pu_2_O_3_ and combining in α-Pu_2_O_3_ to form H_2_. To this end, a comparative analysis is conducted on two diffusion routes: route 1, a single H atom diffuses from site 1–1 to site 2–1; route 2, a single H atom exists after site 2–1, while another H atom diffuses from site 1–1 to site 2–1, and the two H atoms merge to form an H_2_. Use the CINEB method to search for several intermediate transition states in two diffusion processes, as shown in Fig. 10. In the two processes, the energy barriers that H atoms need to cross are 0.59 eV and 0.49 eV, that is, route 2 is more likely to cross than route 1. Transition states occur when atoms diffuse to the vicinity of plutonium, where they need to “squeeze” through the region formed by Pu ions with a certain radius. In route 2, Pu atoms are oxidized to higher valence states due to H atoms acting as oxidants. Pu atoms in higher valence states have radii smaller than those in lower valence states. H atoms are more likely to cross energy barriers and complete diffusion in this process than in route 1. Hydrogen diffuses from PuO_2_ to α-Pu_2_O_3_ in atomic form and combines with other H atoms that diffuse to OVs in α-Pu_2_O_3_ to form H_2_.

### 3.5 Hydrogen dissolution in the PuO_2_/α-Pu_2_O_3_ interface

Hydrogen is dissolved in an atomic state in pure PuO_2_ (fused with O atoms), and can adsorb four H atoms near each O atom, with the adsorption energy gradually increasing. Hydrogen dissolves in a molecular or atomic state in α-Pu_2_O_3_ (captured by OVs), with each OV capable of accommodating four H atoms (which relax and recombine into two H_2_). From PuO_2_ to α-Pu_2_O_3_, the main structure did not undergo significant changes, and the dissolution mode and characteristics did not change either. The interface model composed of PuO_2_ and α-Pu_2_O_3_ also inherits these characteristics.

Similar to the adsorption of a single H atom on the PuO_2_ side of the interface, the maximum number of H atoms accommodated near each oxygen atom is also four. However, the closer to the interface, the lower the energy required for adsorption and the easier for dissolution. On the α-Pu_2_O_3_ side, there is a certain change in the situation. At site 2–1 and site 2–2, closer to the interface, the upper limit of H atoms that can be accommodated in OVs is two (recombining into one hydrogen molecule). As the distance between OVs and the interface increases, their ability to accommodate H atoms increases. Site 2–3 and site 2–4 can accommodate four H atoms (recombining into two hydrogen molecules).

A noteworthy situation has emerged in the structural optimization of various models for hydrogen dissolution at the interface of PuO_2_/α-Pu_2_O_3_. To test the capacity of OVs in α-Pu_2_O_3_ to accommodate H atoms, different numbers of H atoms were placed in each OV. Perform sufficient relaxation, and if there is a sudden change in energy in the structure after relaxation, it is considered to have reached the upper limit for accommodating H atoms. When three H atoms were placed in site 2–1, it was found that in the relaxed structure, two H atoms recombined into H_2_, and another H atom was captured by the O atom at site 1–1. This indicates that hydrogen diffusion within the plutonium oxide phase is not unidirectional. When the hydrogen dissolution in α-Pu_2_O_3_ locally reaches the upper limit while PuO_2_ still has room to accommodate it, there may be a “backflow” of hydrogen.

## 4 Conclusions

The micromechanisms of the hydrogen behaviors in the PuO_2_/α-Pu_2_O_3_ interface are systematically investigated using the first-principles calculations method within DFT schemes.

We find that at the interface, the capture of H atoms by OVs in α-Pu_2_O_3_ is more energetically advantageous than that by oxygen in PuO_2_. The energy required for hydrogen diffusion gradually increases from PuO_2_ to the interface, as well as from the interface to α-Pu_2_O_3_. OVs that already contain H atoms are more conducive to capturing H atoms. The formation of the interface will reduce the capture ability of OVs in α-Pu_2_O_3_. Overall, the types of hydrogen exhibited at the interface are not fundamentally different from those of the two oxides. α-Pu_2_O_3_ is formed by the generation of OVs in PuO_2_ under hypoxia conditions. The interface between PuO_2_ and α-Pu_2_O_3_ acts as a buffer. The behavior differences of hydrogen in different plutonium oxides come from the changes in the atomic environment and ion valence state caused by the OVs. The conclusion of this work supports further understanding of the behavior of hydrogen in plutonium oxides and provides a reference for further research on plutonium corrosion prevention.

## Conflicts of interest

There are no conflicts to declare.

## Supplementary Material
